# An apelin receptor antagonist prevents pathological retinal angiogenesis with ischemic retinopathy in mice

**DOI:** 10.1038/s41598-017-15602-3

**Published:** 2017-11-08

**Authors:** Yuki Ishimaru, Fumiya Shibagaki, Akiko Yamamuro, Yasuhiro Yoshioka, Sadaaki Maeda

**Affiliations:** Department of Pharmacotherapeutics, Faculty of Pharmaceutical Sciences, Setsunan University, 45-1 Nagaotoge-cho, Hirakata, Osaka 573-0101 Japan

## Abstract

Pathological retinal angiogenesis is caused by the progression of ischemic retinal diseases and can result in retinal detachment and irreversible blindness. This neovascularization is initiated from the retinal veins and their associated capillaries and involves the overgrowth of vascular endothelial cells. Since expression of the apelin receptor (APJ) is restricted to the veins and proliferative endothelial cells during physiological retinal angiogenesis, in the present study, we investigated the effect of APJ inhibition on pathological retinal angiogenesis in a mouse model of oxygen-induced retinopathy (OIR). *In vitro* experiments revealed that ML221, an APJ antagonist, suppressed cultured-endothelial cell proliferation in a dose-dependent manner. Intraperitoneal administration of ML221 inhibited pathological angiogenesis but enhanced the recovery of normal vessels into the ischemic regions in the retina of the OIR model mice. ML221 did not affect the expression levels of vascular endothelial growth factor (VEGF) and its receptor (VEGFR2) in the retina. APJ was highly expressed in the endothelial cells within abnormal vessels but was only detected in small amounts in morphologically normal vessels. These results suggest that APJ inhibitors selectively prevent pathological retinal angiogenesis and that the drugs targeting APJ may be new a candidate for treating ischemic retinopathy.

## Introduction

Pathological retinal angiogenesis is caused by the progression of ischemic retinal diseases, such as proliferative diabetic retinopathy and retinopathy of prematurity, and can result in retinal detachment and irreversible blindness. Vascular endothelial growth factor (VEGF) is a primary angiogenic factor that mediates such ischemia-induced retinal neovascularization. Anti-VEGF therapies have substantial therapeutic efficacy^1^. However, VEGF blockade is ineffective in some patients^2^ and it could potentially cause systemic adverse effects^3,4^. In addition, anti-VEGF agents comprehensively suppress retinal angiogenesis and thereby sustain retinal ischemia^5^. Therefore, it is necessary to identify a new drug that more specifically blocks pathological angiogenesis than VEGF inhibitors.

Retinal vascular sprouting under hypoxic conditions is initiated from the veins and their associated capillaries, some of which fail to regenerate the capillary network into the ischemic intraretinal region and form neovascular tufts towards the vitreous^6^. The formation of neovascular tufts is shown to be a consequence of the overgrowth of vascular endothelial cells induced by an overexpression of hypoxia-inducible growth factors^7,8^. Thus, these findings suggest that decreased proliferative signals in endothelial cells of the venules and their associated capillaries under hypoxic conditions could lead to specific inhibition of pathological retinal angiogenesis.

Apelin is an endogenous bioactive peptide ligand for the G protein-coupled receptor APJ^9^. The apelin-APJ system has received attention as a signaling system that has proangiogenic activity under physiological and pathological conditions^10–12^. Apelin expression is induced by hypoxia^13^, and apelin and APJ expressions are increased in the endothelial cells in ischemia tissues^12^. During physiological retinal angiogenesis, apelin is expressed in tip cells, which are motile endothelial cells localized at the leading edge of growing capillaries, and APJ is expressed in stalk cells, which are proliferative endothelial cells that follow behind tip cells and form patent vessels^14,15^. Moreover, APJ are restricted to endothelial cells of the veins and their associated capillaries during retinal angiogenesis^15,16^. We have previously reported that apelin expression is remarkably increased during pathological retinal angiogenesis in an oxygen-induced retinopathy (OIR) mouse model, an ischemic retinopathy model, and that apelin gene deletion markedly reduces pathological retinal angiogenesis^17^. In addition, we found that downregulation of apelin by an intravitreal injection of small interfering RNA (siRNA) increased pericyte coverage of newly formed patent vessels during pathological retinal angiogenesis^18^. Therefore, APJ inhibition could efficiently suppress pathological retinal angiogenesis in ischemic retinopathy.

In this study, we investigated the effect of ML221, a functional small molecule antagonist of APJ^19^, on pathological retinal angiogenesis in the OIR model mice. In our study, we demonstrated that APJ inhibition specifically suppressed pathological retinal angiogenesis in ischemic retinopathy.

## Results

### An apelin receptor antagonist inhibits proliferation of cultured-endothelial cells

**Figure 1:**
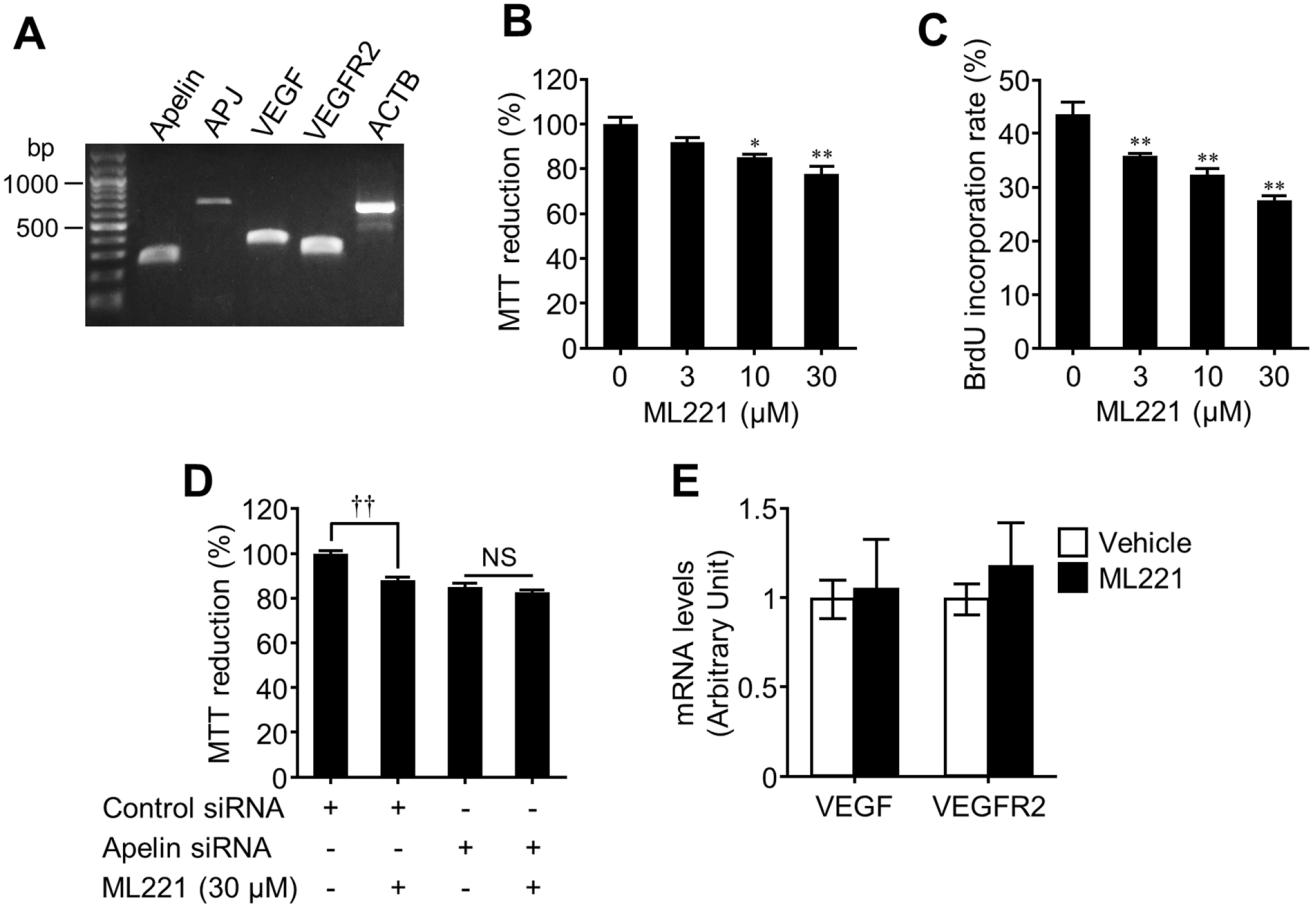
The APJ antagonist inhibits endothelial cell proliferation. (**A**) Representative picture shows that the mRNA expression of apelin (247 bp), APJ (684 bp), VEGF (338 bp), VEGFR2 (285 bp), and ACTB (632 bp) in bEnd.3 cells was examined by RT-PCR. (**B** and **C**) The proliferation of bEnd.3 cells following 24 h incubation with ML221 (0–30 μM) were assessed by the MTT assay (**B**) (n = 4) and the BrdU incorporation assay (**C**) (n = 3). (**D**) bEnd.3 cells were transfected with the apelin siRNA or the control siRNA. After 24 h, the cells were incubated with or without ML221 (30 μM) for 24 h. The cell proliferation was assessed by the MTT assay (n = 3). (**E**) The expression of VEGF and VEGFR2 in bEnd.3 cells at 24 h after exposure to ML221 (30 μM) was analyzed by real-time RT-PCR (n = 5). Data were analyzed by one-way ANOVA with Dunnett’s post hoc test (**B** and **C**), two-way ANOVA with Bonferroni’s post hoc test (**D**) or Student’s t test (**E**) and represent the mean ± SEM. *p < 0.05 and **p < 0.01 vs. 0 μM (ML221), ^††^ *p* < 0.01, NS, not significant.

Since overgrowth of endothelial cells is involved in pathological retinal angiogenesis, we first examined the effect of ML221, an APJ antagonist, on endothelial cell proliferation using mouse microvascular endothelial bEnd.3 cells. We confirmed the expression of apelin and APJ in the cells by end-point reverse-transcription polymerase chain reaction (RT-PCR) (Fig. 1A and Supplementary Fig. 1). The cells also expressed VEGF and VEGFR2 (Fig. 1A). Treatment of ML221 with bEnd.3 cells decreased the 3-(4,5-dimethylthiazol-2-yl)-2,5-diphenyl tetrazolium bromide (MTT) reduction (Fig. 1B) and inhibited the 5′-brom-2′deoxy-uridine (BrdU) incorporation in a dose-dependent manner (Fig. 1C). To confirm whether ML221 inhibits cell proliferation by blocking apelin-APJ signaling, we tested the effect of ML221 on proliferation of the cells transfected with siRNA targeting apelin. Our previous study showed that the apelin siRNA reduced apelin mRNA expression to 5% in the cells^17^. ML221 did not decrease the MTT reduction in the cells transfected with the apelin siRNA (Fig. 1D). Additionally, ML221 did not affect the expression of VEGF and VEGFR2 in the cells (Fig. 1E). These results suggest that ML221 inhibits endothelial cell proliferation by blocking apelin-APJ signaling without affecting the expression of VEGF and VEGFR2.

### The apelin receptor antagonist suppresses pathological angiogenesis in the retina of the OIR model

Next, to investigate the effect of the APJ antagonist on pathological retinal angiogenesis in ischemic retinopathy, we employed the OIR model, which is a widely used system to examine ocular neovascularization, a condition that resembles proliferative diabetic retinopathy in humans^20^. In this model, postnatal day (P) 7 mice are exposed to 75% oxygen for 5 days to cause vascular regression and obliteration in the retina. After P12, the mice are returned to room air and neovascularization occurs toward the avascular area^21^.

**Figure 2:**
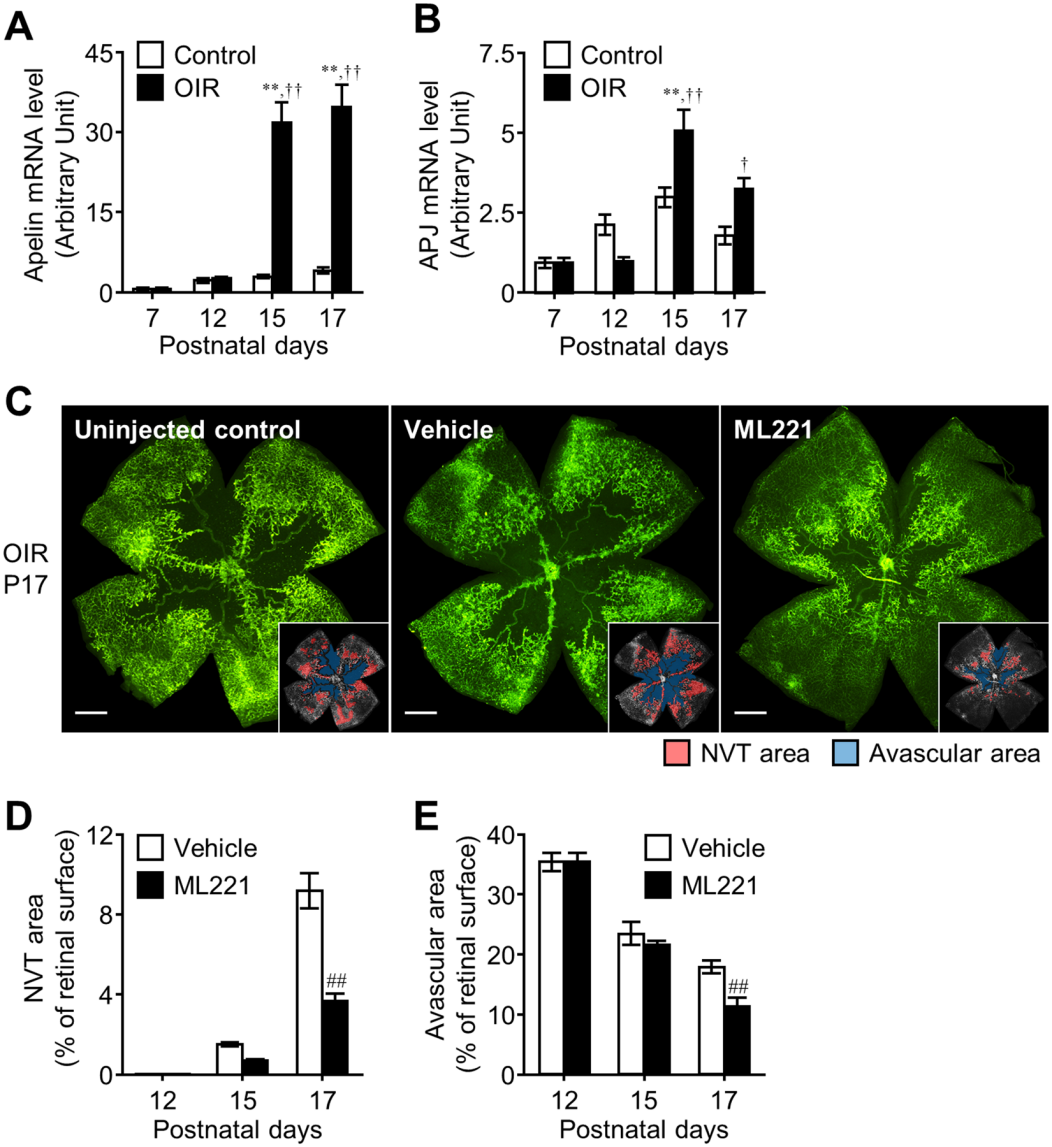
The APJ antagonist suppresses pathological retinal angiogenesis in the OIR model. (**A** and **B**) Apelin (**A**) and APJ (**B**) mRNA expression levels were assessed by real-time-RT-PCR in the retina of the OIR model mice or control mice (n = 3-4). (**C**) Representative pictures show isolectin B4 staining in a retinal flat-mount from the OIR model mice at P17 following intraperitoneal administration of the vehicle or ML221. In the insets, the red area indicates the neovascular tufts (NVT) and the blue area indicates the avascular area. (**D** and **E**) NVT (**D**) and avascular areas (**E**) were quantified (n = 7). Scale bars, 500 μm. Data were analyzed by two-way ANOVA with Bonferroni’s post hoc test, and represent the mean ± SEM. **p < 0.01 vs. Control, ^†^ *p* < 0.05 and ^††^ *p* < 0.01 vs. P7, ^##^ *p* < 0.01 vs. Vehicle.

We confirmed the expression patterns of apelin and APJ in the retina of the OIR model. Consistent with our previous study^17^, apelin mRNA was remarkably elevated in the retina of the OIR model at P15 and P17 in which pathological angiogenesis occurs (Fig. 2A). APJ mRNA expression was also significantly increased during the pathological angiogenic phase in the retina of the OIR model compared with the retina of normally developing mice (Fig. 2B). Isolectin B4 staining revealed that abundant neovascular tufts and the avascular regions emerged in the retina from uninjected mice or vehicle-injected mice with OIR at P17 (Fig. 2C). In contrast, intraperitoneal administration of ML221 markedly reduced the formation of neovascular tufts (Fig. 2C,D). In addition, ML221 enhanced the decrease of the avascular area at P17 (Fig. 2C,E), implying that ML221 facilitated the recovery of normal vessels into the vaso-obliteration area in the retina.

**Figure 3:**
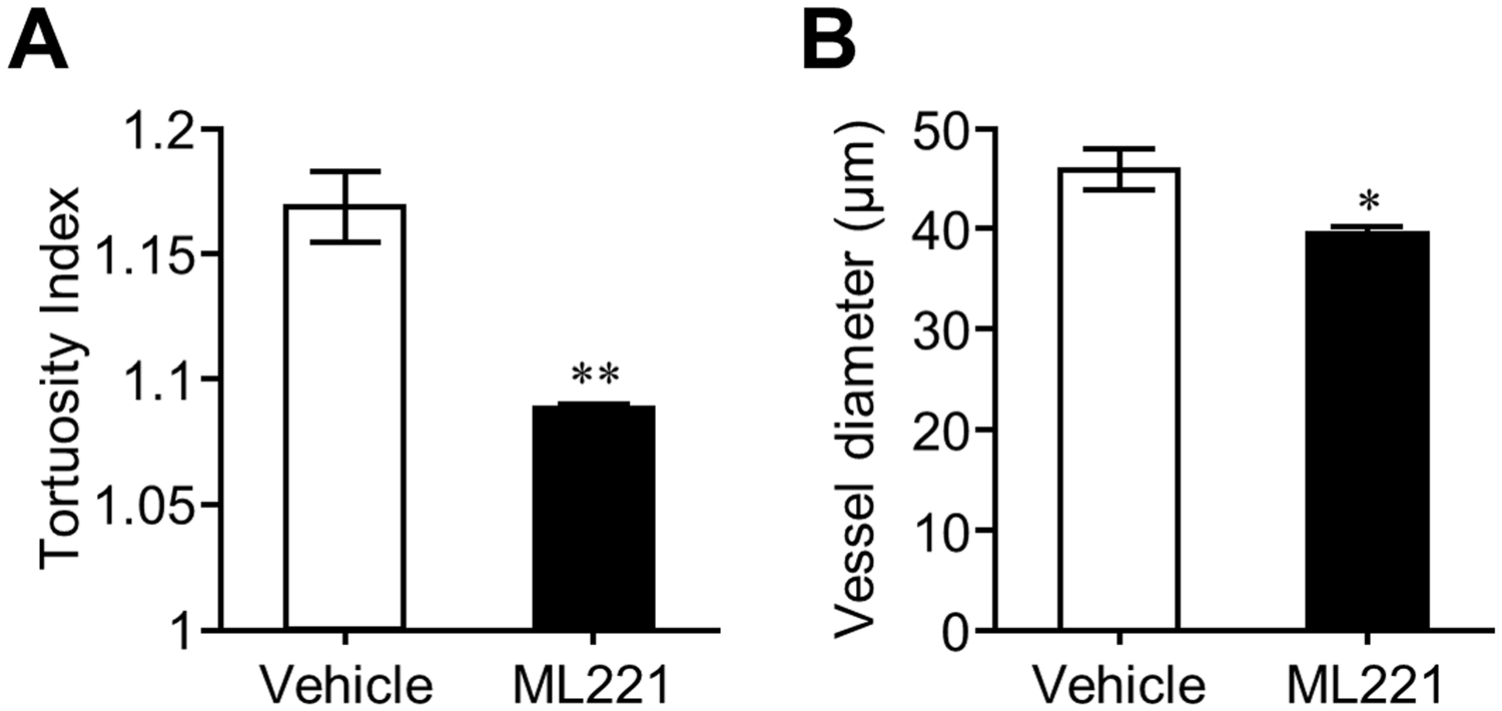
The APJ antagonist reduces vascular tortuosity and dilation in the OIR model. Vascular tortuosity (**A**) and dilation (**B**) in the retina of the OIR model at P17 treated with ML221 or vehicle were quantified (see Material and Methods for details) (n = 7). Data were analyzed by Student’s t test and represent the mean ± SEM. *p < 0.05 and **p < 0.01 vs. Vehicle.

We also evaluated the effect of ML221 on non-neovascular remodeling characterized by tortuosity and dilation of the retinal vessels in the OIR model, because the abnormalities of the retinal vessels are a clinically important finding of ischemic retinopathy in addition to pathological angiogenesis^22,23^. We found that a significant reduction of vascular tortuosity and dilation at P17 in ML221-treated mice compared to vehicle-injected mice (Fig. 3A,B).

**Figure 4:**
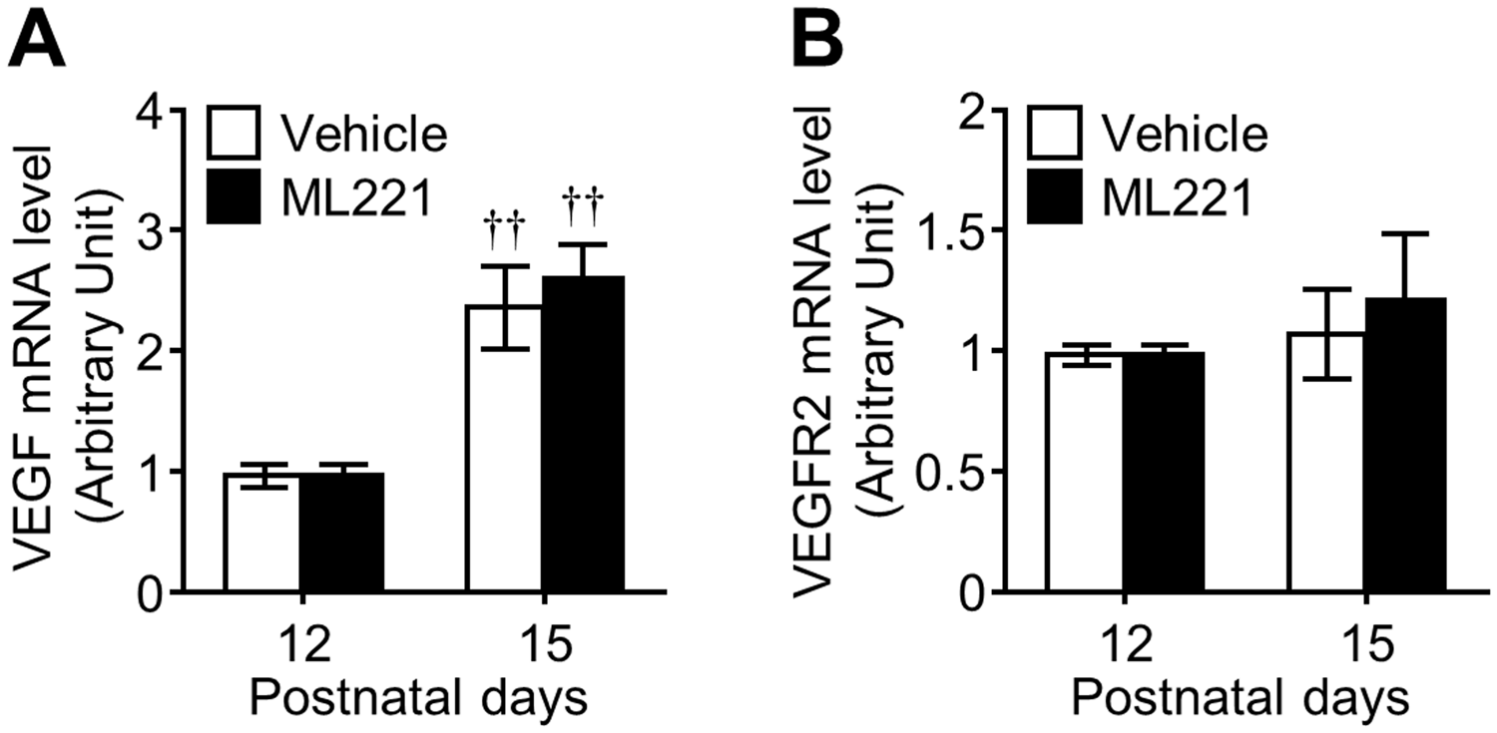
Influence of the APJ antagonist on VEGF and VEGFR2 expressions during pathological angiogenesis in the retina of OIR model. The effect of administration of ML221 on VEGF (**A**) and VEGFR2 (**B**) mRNA expression levels in the retina of the OIR model was examined by real-time-RT-PCR (n = 4). Data were analyzed by two-way ANOVA with Bonferroni’s post hoc test and represent the mean ± SEM. ^††^ *p* < 0.01 vs. P12.

Since VEGF-VEGFR2 axis plays a fundamental role in pathological angiogenesis^24^ and vascular tortuosity and dilation^25^, we then examined whether the effects of ML221 were dependent on the expression of VEGF and VEGFR2. ML221 did not influence the mRNA expression of VEGF and VEGFR2 in the retina of the OIR model at P15 (Fig. 4A,B).

**Figure 5:**
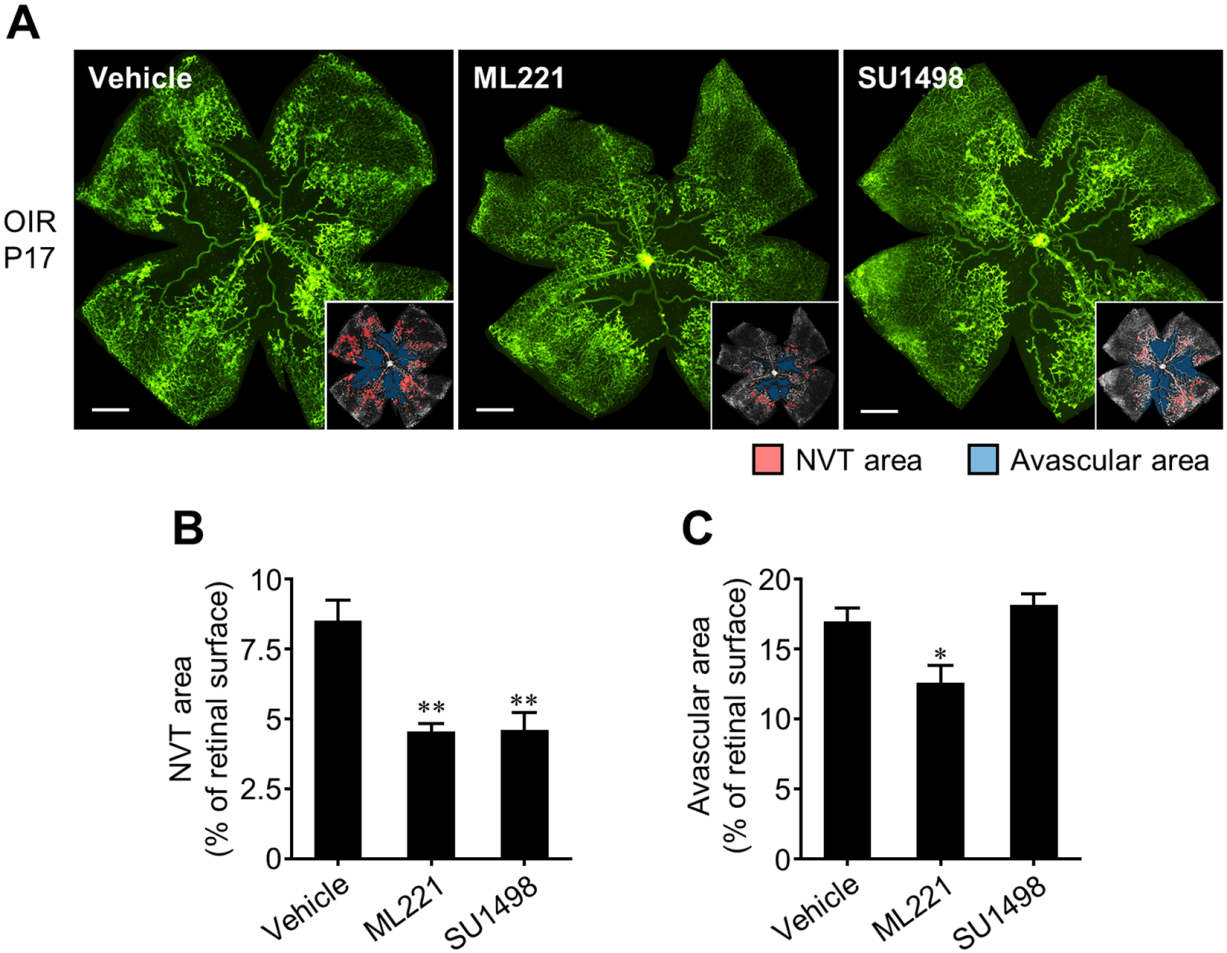
Comparison of the effects of the APJ antagonist with the VEGF inhibitor on retinal angiogenesis in the OIR model. (**A**) Representative pictures show isolectin B4 staining in a retinal flat-mount from the OIR model at P17 treated systemically with vehicle, ML221, or SU1498. In the insets, the red area indicates the neovascular tufts (NVT) and the blue area indicates the avascular area. (**B** and **C**) NVT (**B**) and avascular areas (**C**) were quantified (n = 5). Scale bars, 500 μm. Data were analyzed by one-way ANOVA with Dunnett’s post hoc test, and represent the mean ± SEM. *p < 0.05 and **p < 0.01 vs. Vehicle.

To compare the effects of APJ inhibition and VEGF blockade on the pathological retinal angiogenesis in the OIR model, we administered ML221 or SU1498, a potent and selective inhibitor of VEGFR2 that mediates retinal angiogenesis of VEGF^26^. Intraperitoneal injection of SU1498 markedly suppressed the formation of retinal neovascular tufts (Fig. 5A,B). However, in contrast to ML221, SU1498 did not affect the avascular area compared to vehicle-injected mice (Fig. 5A,C).

### APJ is highly expressed in endothelial cells within the neovascular tufts in the retina of the OIR model

**Figure 6:**
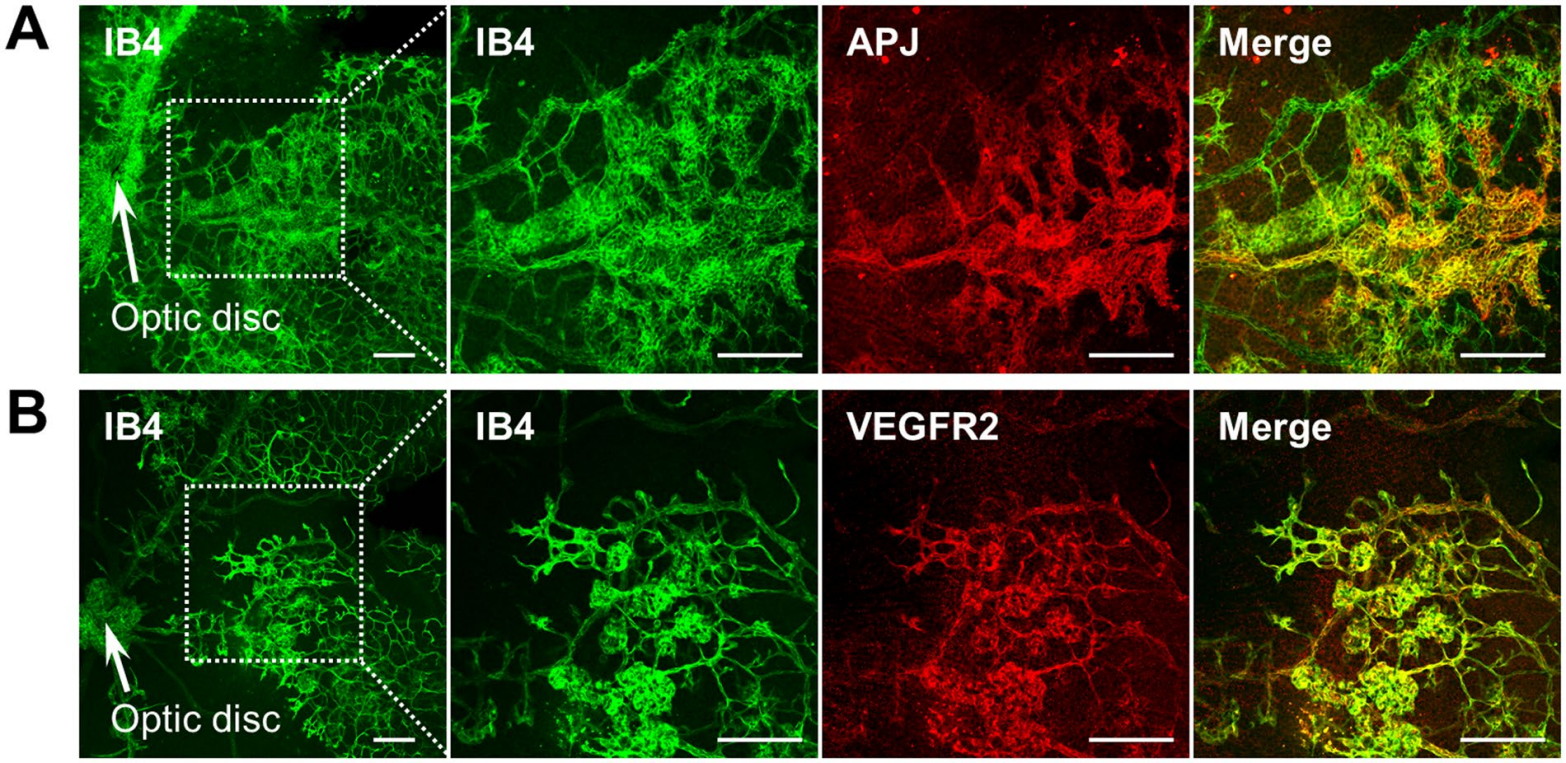
APJ is highly expressed in the endothelial cells within neovascular tufts. Representative pictures show double immunofluorescence of isolectin B4 (IB4; green) and APJ (red) (**A**) or VEGFR2 (red) (**B**) in a retinal flat-mount from the OIR model at P17. These pictures were taken with a confocal laser scanning microscope. Scale bars, 100 μm.

To explore the reason why ML221 specifically inhibited pathological neovascularization, we investigated the distribution of APJ during pathological retinal angiogenesis. Similar to the result of our previous report using the OIR model at P15^17^, immunohistochemical staining revealed that APJ was highly expressed in the endothelial cells within neovascular tufts but was only detected in small amounts in morphologically normal vessels at P17 (Fig. 6A). On the other hand, VEGFR2 was uniformly distributed in the endothelial cells within not only neovascular tufts but also the sprouting edge (Fig. 6B).

## Discussion

We used the mouse model of OIR to assess whether an APJ antagonist could inhibit pathological retinal angiogenesis in ischemic retinopathy. Moreover, we compared the effects of APJ inhibition and VEGF blockade on pathological retinal angiogenesis in this model. We found that ML221, an APJ antagonist, prevented the formation of neovascular tufts, whereas it accelerated revascularization into the avascular area. In contrast, SU1498, a VEGFR2 inhibitor, suppressed the formation of neovascular tufts but did not affect the avascular area, similar to previous reports using a soluble VEGFR1 protein or a VEGFR2 inhibitor^5,27^, suggesting that the VEGF-VEGFR2 axis plays an essential role in not only pathological angiogenesis but also the regrowth of normal vasculature in OIR. Supporting this suggestion, APJ was highly expressed in the endothelial cells within the neovascular tufts but was only detected slightly in morphologically normal vessels, whereas VEGFR2 was ubiquitously distributed in the endothelial cells. These results suggest that APJ antagonists more selectively suppress pathological retinal angiogenesis in ischemic retinopathy than VEGFR2 inhibitors.

The most established therapeutic approach for limiting pathological ocular neovascularization is undoubtedly the blockade of VEGF. However, it has been reported that anti-VEGF treatment is ineffective in some patients^2^ and becomes less effective at blocking vessel growth and at regressing vessels as the neovascularization develops over time^28^. This suggests VEGF-independent proangiogenic mechanisms in the progression of ischemic eye diseases. In the present study, ML221 inhibited pathological retinal angiogenesis and endothelial cell proliferation without affecting the expression of VEGF and VEGFR2, suggesting that the antiangiogenic effect of ML221 is independent of the VEGF-VEGFR2 signaling pathway. This suggestion is in agreement with other studies demonstrating that apelin gene deletion or knockdown reduces pathological retinal angiogenesis without decreasing VEGF expression^17,18^ and that apelin or APJ knockdown inhibits hypoxia-induced endothelial cell proliferation in both the presence and absence of SU1498^13^. Recent clinical studies detected high levels of apelin in the vitreous and the epiretinal fibrovascular membranes of patients with ischemic retinopathy, but these were unrelated to VEGF^29,30^. Another study found high apelin levels in tumor tissues in a VEGF neutralizing antibody non-responder with colorectal cancer in which pathological angiogenesis was associated with the progression^31^, supporting an independent role for apelin and VEGF in pathological angiogenesis. These findings suggest that APJ-targeted therapy may serve as a useful secondary treatment in VEGF inhibitor-resistant patients with neovascular diseases, including ischemic retinopathy.

We observed that ML221 resulted in the decrease of the avascular area in addition to inhibition of the formation of neovascular tufts in the retina of the OIR model, suggesting that ML221 could lead to acceleration of recovery from ischemia. We showed that apelin and APJ expression levels were markedly increased during pathological angiogenesis in the retina of the OIR model compared with the normal retina. Apelin secreted from tip cells binds to APJ in stalk cells and induces their proliferation^14^, suggesting that the overactivation of the apelin-APJ system causes excessive proliferation of the endothelial cells in the retina. The overgrowth of the endothelial cells leads to the formation of neovascular tufts towards the vitreous and the failure of regenerate the capillary network^6^. Apelin or APJ deficiency in mice causes a retardation of physiological retinal angiogenesis^11,14^, implying that the apelin-APJ signaling at the physiological level promotes normal retinal angiogenesis. Therefore, the decrease of the avascular area in ML221-treated mice might be due to the fact that ML221 reduces the apelin-APJ signaling to the proper level that is conducive with normal vascular outgrowth and thereby suppresses pathological angiogenesis and promotes revascularization. However, further investigations are needed to determine the precise molecular mechanisms by which ML221 accelerated revascularization into the avascular area in OIR.

Apelin activates p70 S6 kinase through a PI3K pathway and an ERK pathway mediated via APJ in cultured-endothelial cells and this induces cell division^32^. In addition, apelin- or APJ-deficient retinal tissue during angiogenesis causes a reduction in the phosphorylation of mTOR^14^, which mediates vascular cell proliferation under hypoxia^33^. Moreover, our previous study has demonstrated that apelin deficiency leads to a decrease in retinal endothelial cell proliferation induced by OIR^17^. Taken together with the inhibiting effect of ML221 on cultured-endothelial cell proliferation, we concluded that ML221 prevented pathological retinal angiogenesis by inhibiting endothelial cell division in OIR.

It has been shown that non-neovascular remodeling in the OIR model is characterized by vascular tortuosity and dilation and these vascular changes are a consequence of higher levels of proliferation of endothelial cells^8^. In addition, a recent study reported that apelin was required for non-neovascular remodeling in the retina and demonstrated that apelin gene deletion substantially reduced the emergence of dilated and tortuous vessel phenotypes in another model mouse^34^. Based on these findings, we evaluated the influences of ML221 on tortuosity and dilation of the retinal vessels in the OIR model and found that ML221-treated mice displayed less tortuosity and dilation than vehicle-injected mice. These results suggest that the apelin-APJ system contributes to non-neovascular remodeling in addition to pathological angiogenesis in the OIR model similar to another model and that APJ inhibitors can suppress vascular tortuosity and dilation in OIR.

We previously reported that endogenous apelin protected against N-methyl-D-aspartate-induced retinal ganglion cell death in adult mice^35^. Therefore, to assess the influence of APJ inhibition on the retinal ganglion cells in the OIR model, we counted the number of the ganglion cells in the retinal section obtained from the OIR model treated with ML221. However, ML221 did not decrease the number of the ganglion cells detected with anti-Brn-3a antibody in the OIR model mice at P17 (Vehicle, 117.00 ± 10.52; ML221, 135.87 ± 14.58). This result is supported by our previous observation that apelin deficiency in mice did not affect the number of retinal ganglion cells^35^. Although long term safety studies will be required to determine whether APJ antagonists cause retinal toxicity, our data indicate that intraperitoneal administration of ML221 for 5 days does not influence retinal ganglion cell survival in the OIR model.

Currently, drugs, including anti-VEGF, that are used to treat ischemic retinal diseases are administered through intravitreal injection. However, not only is this mode of treatment painful, but it can also cause serious complications such as endophthalmitis, retinal detachment, intravitreal hemorrhage, and cataract^36^. Therefore, it is necessary to identify an alternative route of administration. In the present study, we assessed the effect of ML221 through intraperitoneal injection and demonstrated the antiangiogenic effect of this agent in the retina. This route of administration would more easily distribute agents systemically than intravitreal injection. Although the systemic influences of ML221 are not known, apelin- or APJ-deficient mice show normal growth after birth^37,38^ and expression of APJ in endothelial cells in the absence of angiogenesis is almost imperceptible^12^. Therefore, APJ inhibitors would be expected to have few side effects even with systemic administration; however, the systemic influences of APJ antagonists require elucidation in future work, because several studies reported that apelin acted on heart contractility^39^ and blood pressure^37^.

In conclusion, our data suggest that APJ antagonists efficiently prevent pathological retinal angiogenesis and thereby lead to vascular regeneration and that the drugs targeting APJ may be a new candidate for treating ischemic retinopathy.

## Materials and Methods

### Cell culture and proliferation assay

Mouse endothelial cell line bEnd.3 cells were maintained in Dulbecco’s modified Eagle’s medium supplemented with 10% heat-inactivated fetal bovine serum. The cells were plated at a density of 2.5 × 10^4^/well in 24 well culture plates for the MTT assay and the BrdU incorporation assay. The MTT assay was performed as described in a previous report^40^. To perform the BrdU incorporation assay, 10 μM BrdU was added to the culture medium. After 2 h, the cells were fixed with 4% paraformaldehyde for 10 minutes. After removing the 4% paraformaldehyde, the cells were incubated with 2 M hydrochloric acid for 10 min at 37 °C for DNA hydrolysis, followed by neutralization with 0.1 M Sodium Borate buffer (pH 8.5) for 30 min at room temperature. Intranuclear BrdU was labeled with mouse anti-BrdU antibodies (BD Biosciences, San Diego, CA, USA). The primary antibody was visualized with biotinylated goat anti-mouse IgG antibodies (DAKO Corp, Carpinteria, CA, USA) plus streptavidin fluorescein isothiocyanate (FITC) (BD Biosciences). Nuclei were detected with Hoechst33342 (Sigma-Aldrich; St. Louis, MO, USA). The BrdU incorporation rate was determined by calculating the numbers of BrdU positive cells per Hoechst positive cells in nine 563.20 μm × 450.56 μm fields of view per sample in scanned images, and the data obtained from each of the nine fields were averaged. Photographs were taken with a fluorescence microscope (AZ-100M, Nikon; Tokyo, Japan). The siRNA experiment was performed as described in a previous report^17^.

### Gene expression analysis

End-point RT-PCR was performed with a Thermal Cycler T100 (BIO-RAD Laboratories, Hercules, CA, USA) and a KOD-FX DNA polymerase (TOYOBO, Osaka, Japan). The sequences of the gene-specific primers for end point RT-PCR were as follows: apelin (forward, 5′-GTTGCAGCATGAATCTGAGG-3′; reverse, 5′-CTGCTTTAGAAAGGCATGGG-3′), APJ (forward, 5′-TGGCTGACTTGACCTTTGTG-3′; reverse, 5′-TGACATAACTGATGCAGGTGC-3′), VEGF (forward, 5′-TTTACTGCTGTACCTCCACCA-3′; reverse, 5′-TGCTGTGCTGTAGGAAGCTCATC-3′), VEGFR2 (forward, 5′-GTGATCCCAGATGACAGCCA-3′; reverse, 5′-AGGTGAGCGCAGTGTGGTC-3′), and β-actin (ACTB) (forward, 5′-GATGGTGGGAATGGGTCAGAAGGA-3′; reverse, 5′-GCTCGTTGCCAATAGTGATGACCT-3′). PCR-amplified products were separated with a 1% agarose gel electrophoresis. Quantitative real-time RT-PCR was performed as previously described^35^. The sequences of the gene-specific primers for real-time RT-PCR were as follows: apelin (forward, 5′-GTTGCAGCATGAATCTGAGG-3′; reverse, 5′-CTGCTTTAGAAAGGCATGGG-3′), APJ (forward, 5′-CCACCTGGTGAAAACTCTCATCA-3′; reverse, 5′-TGACATAACTGATGCAGGTGC-3′), VEGF (forward, 5′- GGAGACTCTTCGAGGAGCACTT-3′; reverse, 5′- GGCGATTTAGCAGCAGATATAAGAA-3′), VEGFR2 (forward, 5′-CGACATAGCCTCCACTGTTTATG-3′; reverse, 5′-TTTGTTCTTGTTCTCGGTGATG-3′), and ACTB (forward, 5′-AGTGTGACGTTGACATCCGTA-3′; reverse, 5′-GCCAGAGCAGTAATCTCCTTCT-3′). Data are expressed as arbitrary units normalized to ACTB expression.

### Animals

All animal studies were performed in accordance with guidelines provided by the Japanese Society for Pharmacology, and the study protocol was approved by the Committee for the Ethical Use of Experimental Animals at Setsunan University, Osaka, Japan. The study was conducted using C57BL/6 N mice. All mice were housed in metallic breeding cages located in a room with a 12 h/12 h light/dark cycle. The humidity was 55%, the temperature was 23 °C, and the mice had free access to food and water.

### Ischemic retinopathy model

An ischemic retinopathy mouse model was produced according to the previously described OIR model^21^. In brief, P7 mice with nursing mothers were exposed to 75% oxygen for 5 days and then placed back in room air.

### Intraperitoneal injection of pharmacological reagents

ML221 (Sigma-Aldrich) dissolved in sterile dimethyl sulfoxide (DMSO) (injection volume 10 mg/kg body weight/day) and SU1498 (LKT Laboratories, Inc.; St Paul, MN, USA) dissolved in sterile DMSO (injection volume 9 mg/kg body weight/day) was administered intraperitoneally daily from P12 to P16 in the mice. Control mice were injected with the same amount of DMSO.

### Immunostaining of whole-mount retina and quantification of vaso-obliteration and neovascularization

Mice were anesthetized with intraperitoneal injection of chloral hydrate (500 mg/kg body weight); they were then perfused with saline and 4% paraformaldehyde in phosphate buffer solution. Enucleated eyes were fixed for 1 hour in 4% paraformaldehyde in phosphate buffer solution, and the retinas were isolated. Retinal cups were exposed to 0.5% Triton-X 100-containing Tris-buffered saline including 1% fetal bovine serum and then stained with FITC-conjugated *Griffonia (Bandeiraea) simplicifolia* isolectin B4 (Vector Laboratories; Burlingame, Calif, USA), rabbit anti-APJ^17^, and rabbit anti-VEGFR2 antibodies (Cell Signaling Technology, Inc.; Danvers, MA, USA). The secondary antibody used was Alexa Fluor 568-conjugated goat anti-rabbit IgG antibody (Molecular Probes Life Technologies; Eugene, OR, USA). The retinas were dissected and flat-mounted with Fluoromont G (Southern Biotech; Birmingham, AL, USA). Photographs were taken with a fluorescence microscope (AZ-100M, Nikon) or confocal laser scanning microscope (FV1000-D, Olympus; Tokyo, Japan). The vaso-obliteration and neovascular tufts areas were quantified by Image-J software [(Wayne Rasband (National Institutes of Health), Bethesda, MD, USA], as previously described^41,42^. Immunohistochemistry for Brn-3a was performed as described in a previous report^35^.

### Evaluation of vascular tortuosity and dilation

Vascular tortuosity and dilation in the retina of the OIR model was assessed as described in a previous report^8^. In brief, the tortuosity in the major vessels of the retina was quantified by tracing a line along the tortuous vessel and comparing it to a straight line traced from the vessel origin at the optic nerve to the first branch point (ImageJ software). To assess vessel dilation, the diameter of the main central retinal vessels was measured at three points (at the point closest to and farthest from the optic nerve and in the middle between these two points) using ImageJ software.

### Statistics

Differences between groups were analyzed using Student’s *t*-test, one-way ANOVA with Dunnett’s post hoc test, or two-way ANOVA with Bonferroni’s post hoc test. *P-*values < 0.05 were considered statistically significant. All results are expressed as the mean ± SEM.

## Electronic supplementary material

Supplementary Information
