# Supplementary material for: An apelin receptor antagonist prevents pathological retinal angiogenesis with ischemic retinopathy in mice
Source: Sci Rep. 2017 Nov 8;7:15062. doi: 10.1038/s41598-017-15602-3 (PMC5678128; doi:10.1038/s41598-017-15602-3)
Supplement: Supplementary file 1 — Supplementary Information [file 41598_2017_15602_MOESM1_ESM.pdf]

Supplementary Information

An apelin receptor antagonist prevents pathological retinal angiogenesis with ischemic retinopathy in mice

Yuki Ishimaru[1,†], Fumiya Shibagaki[1], Akiko Yamamuro[1], Yasuhiro Yoshioka[1], and Sadaaki Maeda[1,*]

[1]Department of Pharmacotherapeutics, Faculty of Pharmaceutical Sciences, Setsunan University, 45-1 Nagaotouge-cho, Hirakata, Osaka 573-0101, Japan

*_Corresponding author_
Sadaaki Maeda, Ph.D.
Department of Pharmacotherapeutics, Faculty of Pharmaceutical Sciences, Setsunan University
45-1, Nagaotouge-cho, Hirakata, Osaka 573-0101, Japan
Tel: +81-72-866-3147
FAX: +81-72-866-3146
Email: smaeda@pharm.setsunan.ac.jp

†_Co-corresponding author_
Yuki Ishimaru, M.S.
Department of Pharmacotherapeutics, Faculty of Pharmaceutical Sciences, Setsunan University
45-1, Nagaotouge-cho, Hirakata, Osaka 573-0101, Japan
Tel: +81-72-866-3132
FAX: +81-72-866-3146
Email: ishimaru@pharm.setsunan.ac.jp

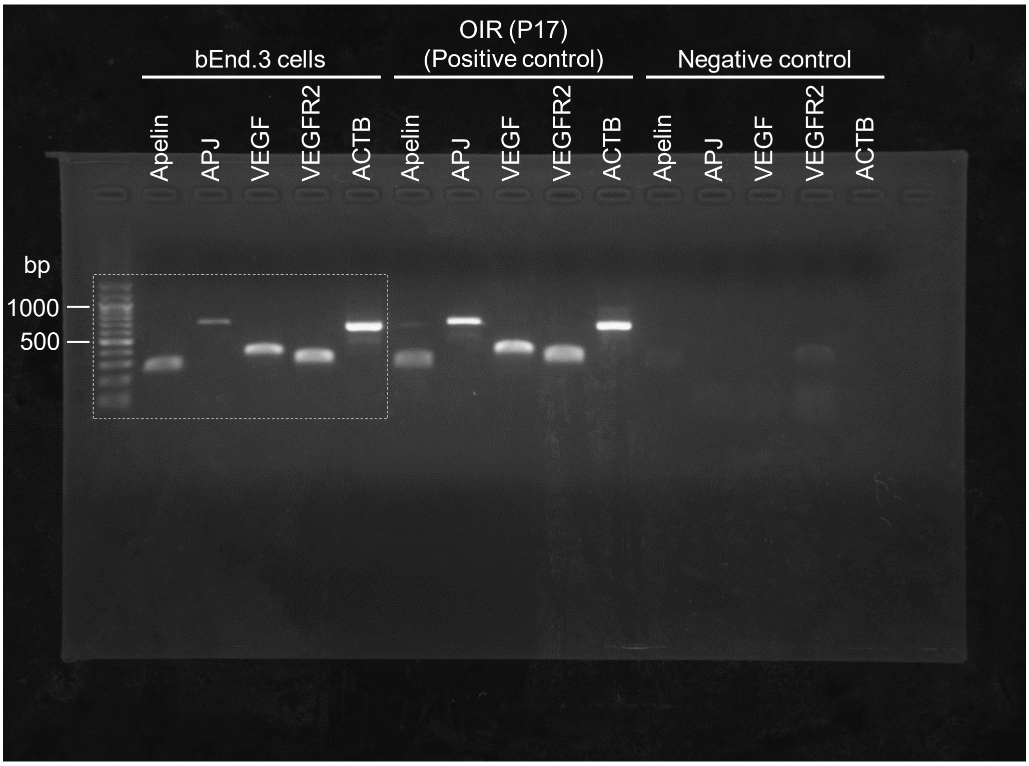

Supplementary Figure 1. RNA expressions analysis. This picture shows the full-length gel image for Fig. 1A. The boxed area in the picture is shown in Fig. 1A.
